# Intelligent robot-assisted fracture reduction system for the treatment of unstable pelvic fractures

**DOI:** 10.1186/s13018-024-04761-5

**Published:** 2024-04-30

**Authors:** Zhengjie Wu, Yonghong Dai, Yanhui Zeng

**Affiliations:** The Eighth Clinical Medical College of Guangzhou University of Chinese Medicine, Foshan, Guangdong China

**Keywords:** Navigation, Pelvic fracture, Closed reduction, Elastic traction, Robot-assisted surgery

## Abstract

**Background:**

Precise and minimally invasive closed reduction is the premise of minimally invasive internal fixation. This paper aims to explore the safety and efficacy of a robot-assisted fracture reduction system (RAFR) in the treatment of pelvic fractures and to analyze its clinical advantages and existing problems.

**Methods:**

The RAFR system intelligently designed the optimal reduction path and target position based on a preoperative three-dimensional(3D) CT scan of the patient. The reduction robotic arm automatically reduced the affected hemipelvis according to the pre-planned reduction path.

**Results:**

The average residual displacement was the 6.65 ± 3.59 mm. According to Matta’s criteria, there were 7 excellent, 10 good, and 3 fair, and the excellent and good rate was 85%. No postoperative complications occurred.

**Conclusion:**

In our study, the RAFR system could complete accurate and minimally invasive closed reduction for most patients with unstable pelvic fractures, which could achieve good fracture reduction quality and short-term efficacy.

## lntroduction

Pelvic fracture accounts for 2%∼8% of all fractures, which can be seen in various high energy and low energy injuries, various displacement modes, often combined with abdominal and pelvic organ injuries and massive bleeding. Among all fractures, the disability rate and mortality rate are the highest, the disability rate is about 60%, and the mortality rate reaches 8%∼14% [1–8]. Accurate minimally invasive closed reduction is the basis of minimally invasive treatment of unstable pelvic fractures [9, 10]. Studies have shown that poor fracture reduction is closely related to the failure of internal fixation, fracture malunion, persistent postoperative pain, gait change and walking difficulties, which will seriously affect the work and life of patients, and improving the quality of reduction can bring better functional results [11–19].

Open reduction, freehand closed reduction, and frame-assisted reduction are all overly dependent on the surgeon’s experience and technique. Open reduction is associated with elevated levels of damage, higher blood loss, extended recovery time, lower satisfaction with reduction outcomes, and elevated rates of complications and disability [20, 21]. Conventional fluoroscopy-guided freehand closed reduction is only applicable to fractures with minimal displacement and relatively simple fractures. In addition, the current reduction frames are too bulky to achieve multi-dimensional precise reduction operations. With the development of robotics, robot-assisted treatment of pelvic fractures is increasingly popular, but it mainly focuses on pelvic fracture fixation, less involving robot-assisted fracture reduction [22–24]. At present, there is no intelligent serial RAFR system based on 3D image guidance that can be truly used for minimally invasive reduction of complex pelvic fractures in clinical practice [8, 25].

In this study, we used the world’s first and only intelligent RAFR system designed for minimally invasive reduction of complex pelvic fractures, which was granted a Class III medical device registration certificate by the National Medical Products Administration (NMPA) of China on December 11, 2023. The research included 20 patients with unstable pelvic fractures who underwent robot-assisted minimally invasive closed reduction and percutaneous screw internal fixation. The objective of this study is to analyze the safety and effectiveness of the RAFR system and to investigate its key technologies and existing problems.

## Methods

### Inclusion and exclusion criteria

The study protocol received approval from our Institutional Ethical Review Committee (No: QX[2022]001), and informed consent was obtained from all participants. This retrospective analysis involved patients between June 2022 and November 2023. The procedures, including robotic-assisted closed reduction and internal fixation of pelvic fractures, were conducted at the first author’s institution by a consistent surgical team with extensive clinical expertise.

Inclusion criteria were as follows: (1) closed unstable pelvic fractures (Tile types B or C) necessitating reduction; (2) patients who were fully informed about the study’s benefits and risks, and who voluntarily agreed to participate by providing informed consent.

Exclusion criteria were defined as follows: (1) presence of severe open injuries or rupture of the abdominal pelvic cavity and organs with wound contamination; (2) bilateral pelvic fractures with significant displacement; (3) unstable vital signs or inability to undergo anesthesia or surgery; (4) poor local skin condition or infection at the intended implant site or surrounding soft tissue; (5) presence of metal fixators in the CT imaging area or severe obesity affecting image acquisition; (6) presence of systemic diseases such as severe cardiac, hemorrhagic, or respiratory conditions; (7) pathological fractures, including primary or metastatic tumors; and (8) stable pelvic ring injuries (Tile type A).

### Preoperative preparation

Anteroposterior (AP), inlet, and outlet views of the pelvis were obtained and complemented with CT scans. Supracondylar femoral traction was applied to patients with vertical instability. The surgical plan was devised considering the fracture type, anticipated reduction outcome, and the expected feasibility of creating bony tunnels.

### Introduction to the intelligent RAFR system

**Fig. 1:**
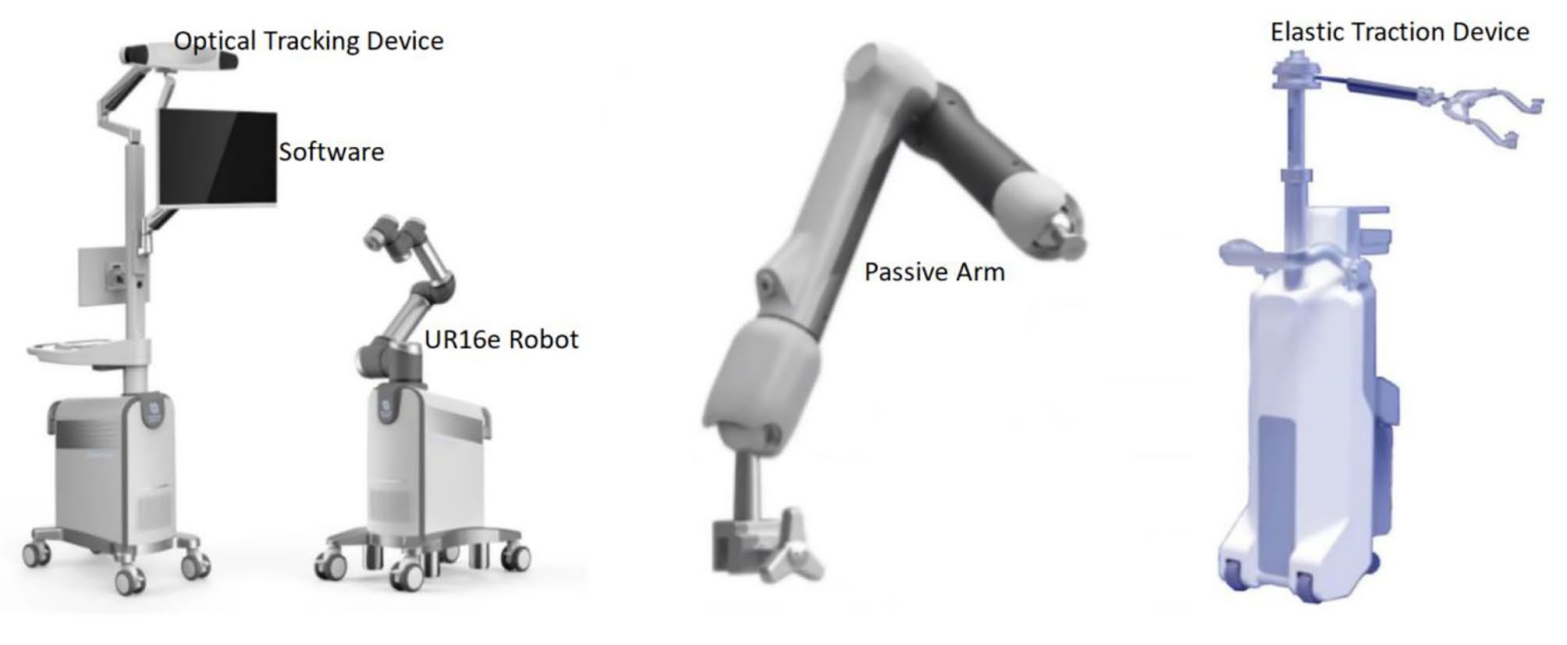
Composition of the intelligent RAFR system

The RAFR system is comprised of five primary components, as illustrated in Fig. 1: pelvic fracture reduction software (encompassing reduction path planning, intraoperative navigation, and registration software), an optical tracking device (NDI Polaris Vega ST and trackers), a reduction robot (UR16e), a holding device affixed to the unaffected hemipelvis, and an elastic traction device. The optical tracking device was linked to the patient’s pelvis and the robot, facilitating real-time tracking throughout the reduction procedure. The holding device is constituted by two nine-degree-of-freedom electrically controlled passive arms, connected to the operating table via a U-shaped base, ensuring secure stabilization of the unaffected hemipelvis. Positioned at the foot end of the operating table, the elastic traction device was installed to counterbalance the restraining forces exerted by the soft tissues surrounding the pelvis during the reduction process [26].

### The preoperative preparation of the RAFR system

**Fig. 2:**
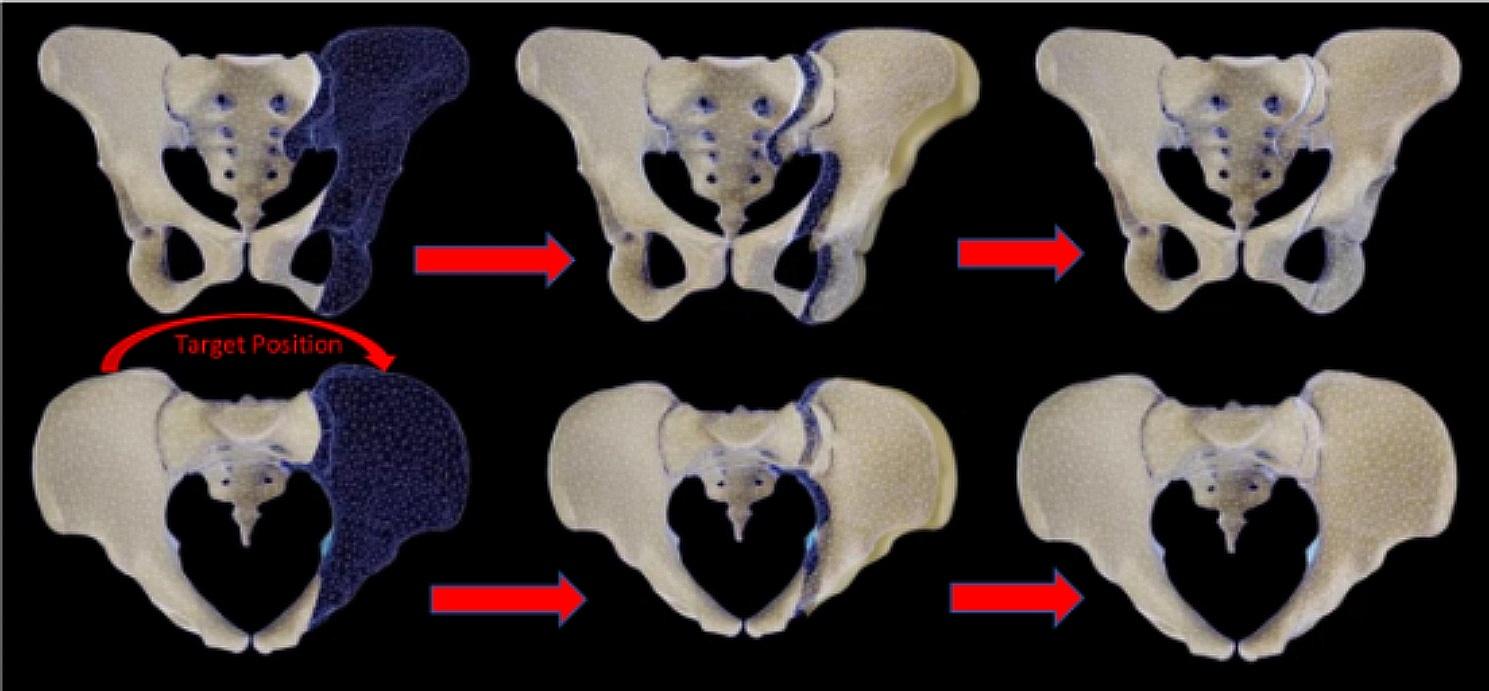
Schematic diagram of the intelligent reduction planning process after mirror matching

The preoperative CT data were integrated into the robotic system, and the pelvic fracture images were segmented into the unaffected and affected sides using planning software. Adhering to the principle of mirror symmetry, the mirror model on the unaffected hemipelvis was considered representative of the anatomical position of the affected hemipelvis. The pelvic shape was meticulously constructed using more than 90,000 points [27] (Fig. 2). The RAFR system employs a proprietary automatic reduction algorithm based on the self-developed shortest path planning methodology. Leveraging a comprehensive mirror hemipelvic reconstruction template, the software autonomously executes optimal reduction path planning [28]. Following the completion of planning, the physician conducts a comprehensive final review of the planning outcomes. By previewing the reduction path, potential collision positions are identified, and adjustments to the reduction path can be made if deemed necessary. To prevent blockage, collisions, and locking during fracture reduction, one or more path points may be strategically set. Subsequently, the reduction simulation is reiterated to guarantee the safety and seamless execution of the reduction path.

### Surgical procedure

**Fig. 3:**
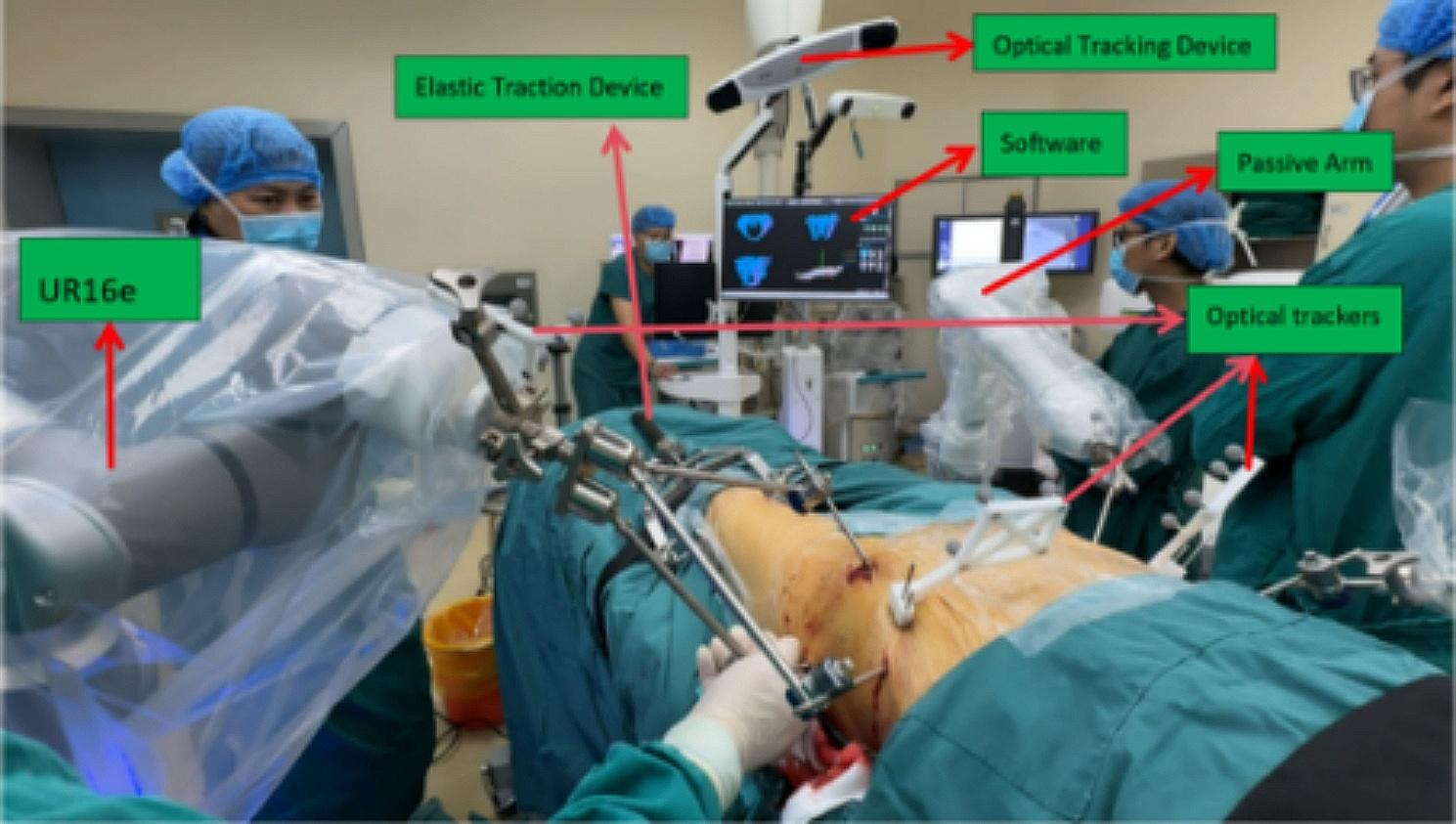
Spatial position of the RAFR system in the operating room

**Fig. 4:**
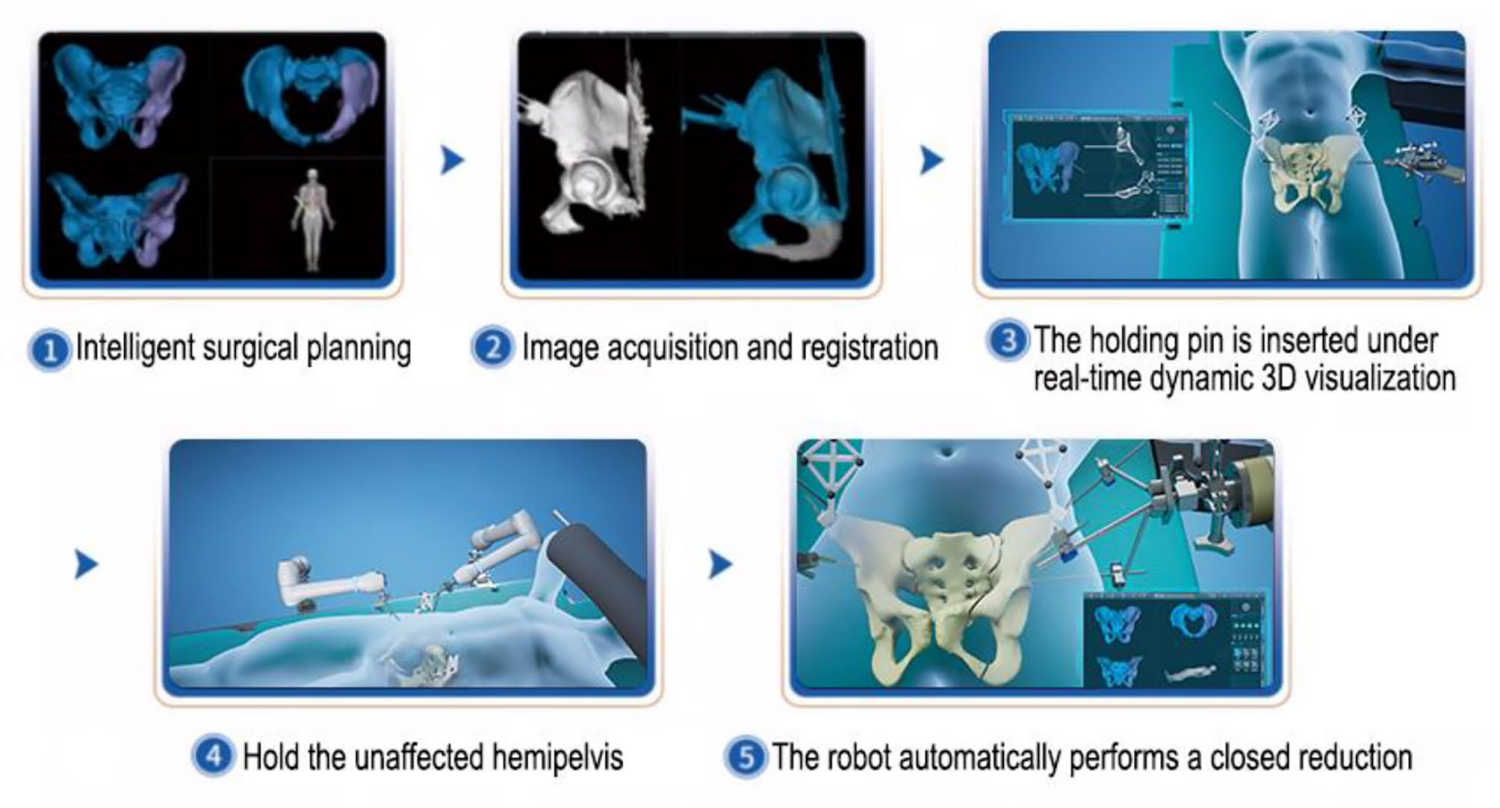
Flow chart of the core surgical procedures

**Fig. 5:**
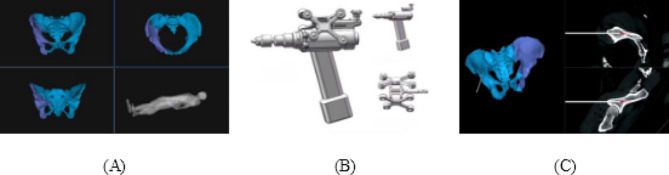
(**A**) After image registration, the position of the pelvis can be tracked in real time and displayed on the screen. (**B**) The structural composition of the navigated hand drill. (**C**) Real-time 3D visualization of the position and depth of the holding pin

**Fig. 6:**
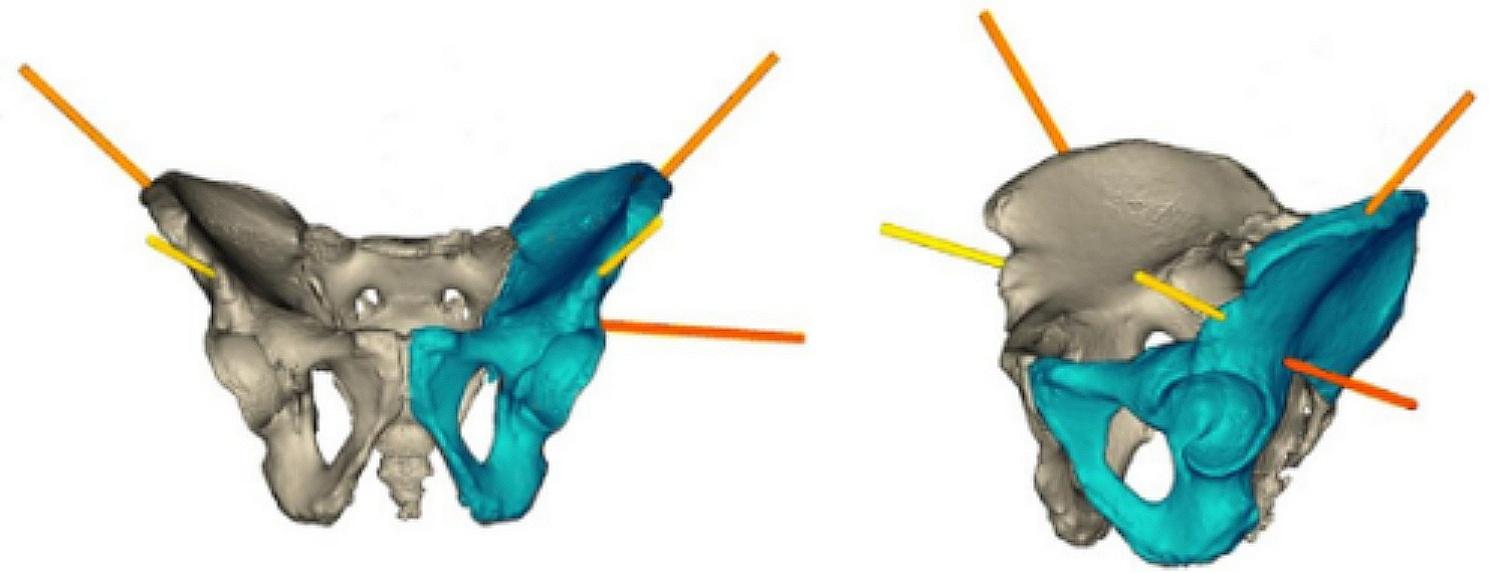
The positions of the five Schantz pins

- The patient was under general anesthesia and laid supine on the full-penetration operation bed. The hip pad was high for disinfection and nails.
- The spatial position of the RAFR system in the operating room is shown in Fig. 3. Core surgical procedures are shown in Fig. 4.
- Two passive holding arms were connected to the pelvic operating bed and the front connector was installed. Routine disinfection and napkin laying.

d. The patient tracer was stably mounted on the bilateral anterior superior iliac spine, and the tracer was also mounted at the end of the robotic arm.
e. The CBCT data of the bilateral pelvis and trackers were collected and transmitted to the main console computer system, and registered with the preoperative 3D CT image. The registration coincidence rate should reach more than 80%. During image registration, two passive arms and the reduction robotic arm were covered with sterile protective sleeves to establish a sterile working environment.
f. After image registration, use the hand drill navigation function(Fig. 5) to place five Schantz pins in the optimal position [29](Fig. 6). The Schantz pins on the unaffected side were connected and fixed to the passive holding arm to ensure the stability of the unaffected side during the reduction process. The Schantz pins on the affected side were connected to the end of the reduction robotic arm to manipulate the movement of the affected hemipelvis.
g. Supracondylar femoral traction was performed in patients with pelvic fractures with vertical displacement. Placed the elastic traction device on the affected side of the tail of the bed and connected the femoral traction needle to the traction device.
h. The reduction robotic arm moved the affected hemipelvis in a gradual and stable manner according to the pre-planned reduction path until the target position was reached.
i. The robotic arm is automatically locked to maintain the pelvic position, and the reduction results were then evaluated by fluoroscopy.
j. After successful reduction, the implant selection is mainly based on the fracture type. The posterior ring of the pelvis was fixed using percutaneous sacroiliac joint screws. The anterior pelvic ring may be fixed with front column screws, an external fixation support, or an INFIX system.
k. Standard pelvic anterior and posterior and inlet X-rays were taken before the end of the procedure to ensure satisfactory reduction and correct placement of channel screws.
l. After the verification, the lower skin layer and skin were sutured, and the operation was completed.

### Typical cases

**Fig. 7:**
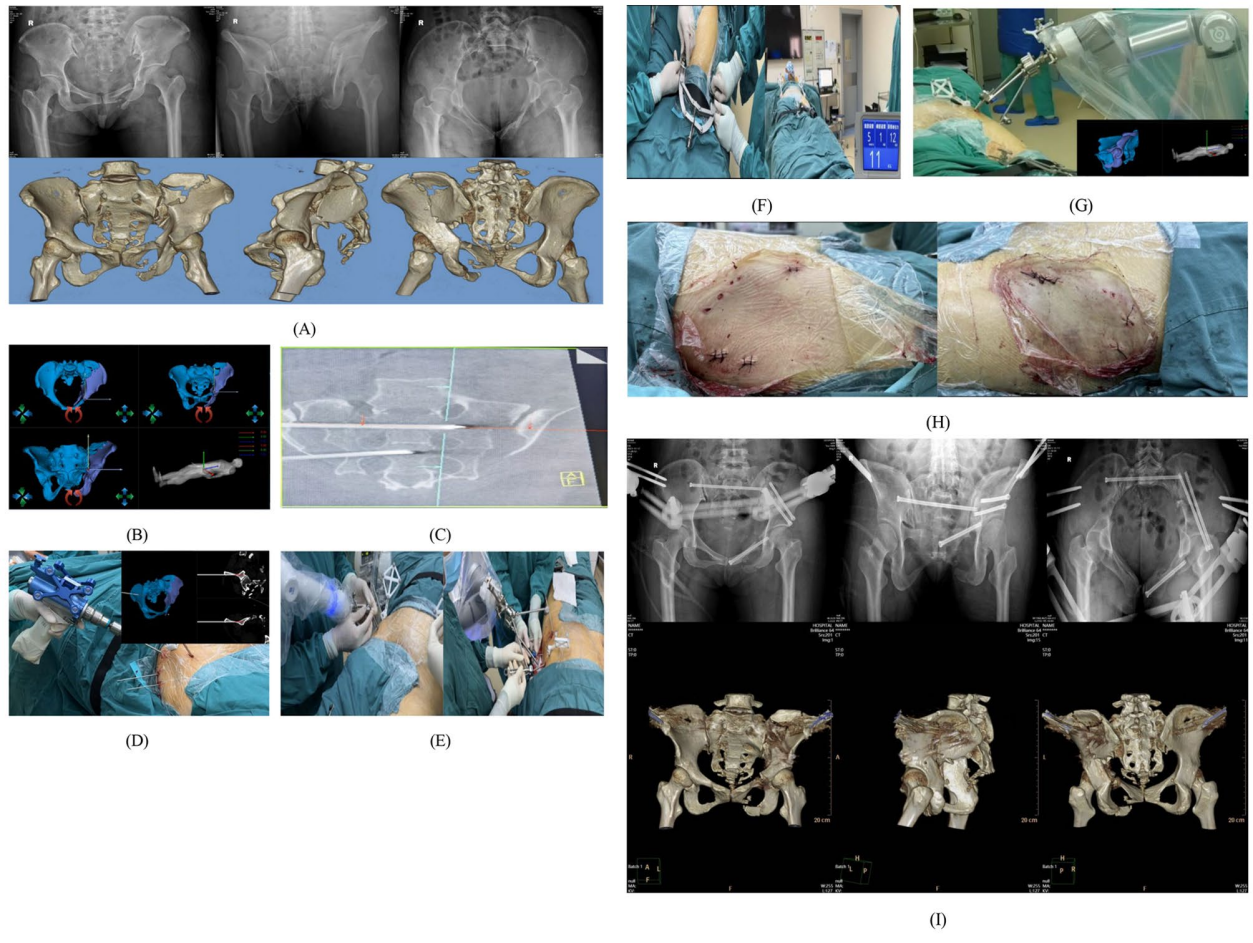
Case Presentation: (**A**) Preoperative X-ray and 3D CT images depict the patient’s status. (**B**) Subsequent to preoperative planning of the reduction path, fine-tuning of the path and target position was executed. (**C**) Two sacroiliac screw guide pins were pre-placed from the unaffected side of the posterior pelvic ring. Post-reduction, these guide pins could directly penetrate into the sacroiliac joint of the affected side, facilitating temporary fixation of the posterior ring. Alternatively, they could be directed through the skin on the affected side of the pelvis to guide sacroiliac screw fixation of the posterior ring. (**D**) The Schantz pin was inserted under real-time 3D navigation. (**E**) Connection between the five Schantz pins and the holding device was established. (**F**) The femoral condylar traction pin was linked to the elastic traction device. (**G**) Supervised by real-time 3D navigation, the robotic arm autonomously moved the affected hemipelvis along the pre-planned reduction path, achieving autonomous reduction. (**H**) Skin incisions. (**I**) Postoperative X-ray and 3D CT images illustrate the patient’s outcome

A 37-year-old female presented with a type Tile C2 pelvic fracture accompanied by a L1 vertebral burst fracture, sternobody fracture, left coracoid fracture of the left scapula, bilateral multiple rib fractures, left proximal radius fracture, left calcaneal fracture, left ankle fracture with ankle subluxation, and posterior dislocation of the left elbow. On the 19th day following admission, the minimally invasive closed reduction operation was performed utilizing the RAFR system. Following reduction, one pubic branch retrograde screw, one LC-2 screw, and one sacroiliac joint screw were inserted to restore the overall normal shape of the pelvic ring, complemented by an external pelvic fixation stent to reinforce pelvic ring stability(Fig. 7).

### Evaluation indicators

After surgery, X-rays were taken and CT scanned. According to the Matta’s criteria, the reduction quality of fracture is determined by the maximum displacement (≤ 4 mm is excellent, 5 ∼ 10 mm is good, 10 ∼ 20 mm is fair, > 20 mm is poor) [12, 30–34]. All patients were observed for any complications such as screw loosening, surgical port infection, iatrogenic fracture, and vascular nerve injury.

### Statistical analysis

In this study, the normality of all continuous count data was assessed using a one-sample Kolmogorov-Smirnov test. Descriptive statistics, including mean (standard deviation), were employed for data conforming to a normal distribution, while median (lower quartile, upper quartile) was used for non-normally distributed data.

Statistical descriptions were performed using IBM SPSS Statistics 26.

## Results

A total of 20 subjects, comprising 11 males and 9 females, participated in the study, with an age of 49.25 ± 19.90 years and a body mass index of 22.69 ± 1.98 kg/m^2^. Among them, 17 patients exhibited concomitant fractures or organ injuries. Based on the Tile typing criteria, 5 patients were classified as type B, while 15 patients were classified as type C. The interval between injury and surgery was 12(10,14) days.

During the procedure, the number of fluoroscopy was 29.5(18.5,58.75) times, with a fluoroscopy time of 25.5(16.65,35.25) seconds. The reduction time was 58.5 ± 3.36 min, and the operation time was 206(200.75,211.5) minutes. The volume of bleeding was 100(62.5,200) ml, and the residual displacement measured 6.65 ± 3.59 mm.

According to the Matta’s criteria for fracture reduction, of the 20 cases, 7 were excellent, 10 were good, 3 were fair, and the excellent and good rate was 85%. None of the patients had any postoperative complications.

## Discussion

In light of the widespread adoption of the minimally invasive approach within the orthopedic domain, closed reduction complemented by minimally invasive percutaneous instrumentation has progressively emerged as the preferred strategy for managing pelvic fractures. However, the intricate nature of the pelvic structure poses challenges during the reduction process, thereby imposing limitations on the broader clinical application of minimally invasive treatments for pelvic fractures [35]. In this study, the only intelligent serial RAFR system based on 3D image guidance that can be used for minimally invasive reduction of complex pelvic fractures is adopted as the treatment method, which truly realized the intelligent and minimally invasive surgery of pelvic fracture reduction.

### Analysis of the efficacy and safety of the RAFR system

In previous study conducted by our surgical team [36], the treatment results of 112 patients with unstable pelvic fractures treated with freehand closed reduction exhibited the following metrics: an average intraoperative blood loss of 243 ml, an average operation time of 197.91 min, an excellent and good reduction rate of 85.71%. The incidence of postoperative complications was 11.61%.

In this study conducted by our surgical team, mean intraoperative blood loss, mean operation time, excellent and good reduction rate, and incidence of postoperative complications were 133.25 ml, 204.75 min, 85% and 0%, respectively.

Compared with the previous surgical method of our surgical team, the new surgical method has significantly reduced the average intraoperative blood loss, the average operation time is slightly longer, the excellent and good reduction rate is similar, and the incidence of postoperative complications is significantly reduced. In this study, a 100% reduction success rate was achieved using the RAFR system, which was not possible with freehand closed reduction during our previous surgery. In the early stages, the relatively longer reduction time, fluoroscopy time and operation time were attributed to our surgical team’s unfamiliarity with the RAFR system. As a new surgical method, we consider it acceptable.

The RAFR system, based on 3D image guidance, treats the pelvis as a 3D entity for reduction planning and operation. After achieving the target reduction positions in both anterior and posterior pelvic rings, fixation is performed to avoid mutual interference. Real-time observation of fracture reduction reduces judgment errors. The intelligent design of the RAFR system reduces technical and personnel requirements, saving on learning costs and human resources. Thanks to the repeatability of the robot, the uniformity of surgical quality has significantly improved.

Safety represents the paramount concern for medical devices. The RAFR system can achieve zero radiation exposure for surgeons during surgery. The system developed a verification procedure for image registration. Only when the registration accuracy is at least greater than 80%, can the next reduction procedure be carried out, which improves the success rate and safety. Real-time dynamic three-dimensional visualization navigation can accurately determine and control the direction, angle, and depth of the holding pin throughout the process, safely establish a screw channel, and reduce the risk of holding-pin loosening and even iatrogenic bone fractures. The reduction robotic arm boasts a maximum load capacity of 160 N, which is less than the force typically exerted by a surgeon’s freehand manipulation in conventional surgery. Moreover, it offers synergistic force-position control, ensuring the safety of the reduction operation. The passive holding arm is equipped with a lock to prevent accidental unlocking while in a locked state. The elastic traction device is equipped with a one-dimensional force sensor, which ensures that the traction force does not exceed the safety value. When software or hardware malfunction occurs, the reduction robot will automatically stop, and the surgeon can also manually perform an emergency stop to ensure the safety of the surgery.

### Key technologies used by the RAFR system

The overall working principle of this system is designed based on the principle of mirror symmetry of pelvic structure [28, 37–41], and allow the operator to manually adjust the planning error. Due to the limited possibility of obtaining morphological data of the affected hemipelvis prior to fracture in clinical practice, using pelvic symmetry reduction method is more closely aligned with clinical reality. In addition, the RAFR system supports the surgeon to set up and save multiple reduction path points on the basis of computer automatic planning to ensure a smooth reduction process.

Accurate registration of preoperative 3D CT images and intraoperative real-time images is the prerequisite of precise reduction. The RAFR system adopts the new registration mode of preoperative CT and intraoperative CBCT, and realizes dynamic three-dimensional real-time navigation through cross-modal image registration and fusion. The system uses near-infrared (IR) light to locate the position of the tracer placed on both sides of the pelvis. The collected measurement data is of sub-millimeter accuracy and repeatable, which can conduct dynamic three-dimensional real-time navigation, and realize the full visualization of the closed reduction path [8]. The next reduction procedure occurs only if the registration accuracy is at least greater than 80%. Compared to the conventional methods [42–44], The RAFR system does not require pre-surgical implant markers, thereby reducing the risk of pain and infection.

In the case of closed reduction, the stability of the pelvis is critical. The passive holding arms of the RAFR system can easily and rigidly connect to the orthopedic operating bed and the holding pins. The unique design of the U-shaped base keeps it away from the surgical area to facilitate disinfection and paving, while avoiding interference with reduction and fixation operations. In the locked state, the robotic arm can load up to 24 kg, providing powerful fixation of the pelvis on the unaffected side and assisting in the accurate reduction of the fracture fragments on the affected side. On the basis of the original two holding pins, a horizontal holding pin was added to the top of the acetabulum to fix the pelvis on the affected side more stably. Under the guidance of 3D visualization, the holding pins are placed in the area with rich bone mass to improve the stability, which is conducive to the robot to stabilize the pelvis and efficiently exert the reduction force. This reduces the risk of needle loosening and iatrogenic fractures.

The surgeon usually needs a force greater than 200 N in pelvic reduction surgery, while the maximum force of commonly used medical robotic arms is only 160 N, which cannot reach the force required for reduction. The RAFR system effectively reduces the requirement for reduction force through elastic traction, solving the issue of insufficient reduction force associated with existing robotic arms and improving the reduction success rate. The lower limb elastic traction device of the RAFR system balances the resistance generated by the muscles and ligaments to improve the flexibility of reduction operation [45]. We found in clinical practice that the mean reduction operating force was decreased by approximately 35% and 58% under 5 kg and 10 kg elastic traction compared to rigid traction, respectively.

### Shortcomings of the RAFR system

In clinical applications, the RAFR system has some limitations. The reduction path planning is based on pelvic symmetry, making it challenging to provide precise and effective reduction path planning for bilateral pelvic fractures with abnormal morphology or severe comminution on the unaffected side. If both ilium are too comminuted to firmly place the holding pins, surgery would be difficult to proceed as well. If the iliac bones on both sides are excessively comminuted, thereby precluding the stable placement of holding pins, the surgical procedure also becomes impracticable. The RAFR system lacks the ability to automatically adjust the reduction path, and it is unable to automatically unlock when encountering bony obstruction during the reduction process. Although optical positioning systems possess high-precision locational capabilities, they are susceptible to interference from objects within the surgical field, thereby impacting the accuracy and timeliness of tracking.

## Conclusion

In our study, the RAFR system could complete accurate and minimally invasive closed reduction for most patients with unstable pelvic fractures, which could achieve good fracture reduction quality and short-term efficacy. The RAFR system, in a data-driven manner, replicates expert experience, providing technological support for the intelligent, precise, minimally invasive, and homogeneous development of pelvic fracture treatment. Finally, since our hospital is one of the clinical trial sub-centers, there are relatively few cases, and we will conduct randomized controlled trials to further verify the efficacy and safety of the new Surgical method if conditions permit.

## Data Availability

No datasets were generated or analysed during the current study.

## Abbreviations

3D: Three-dimensional
RAFR System: Robot-assisted fracture reduction system
